# Supplementary figures and images for: Assessing genomic diversity and signatures of selection in Original Braunvieh cattle using whole-genome sequencing data
Source: BMC Genomics. 2020 Jan 8;21:27. doi: 10.1186/s12864-020-6446-y (PMC6950892; doi:10.1186/s12864-020-6446-y)

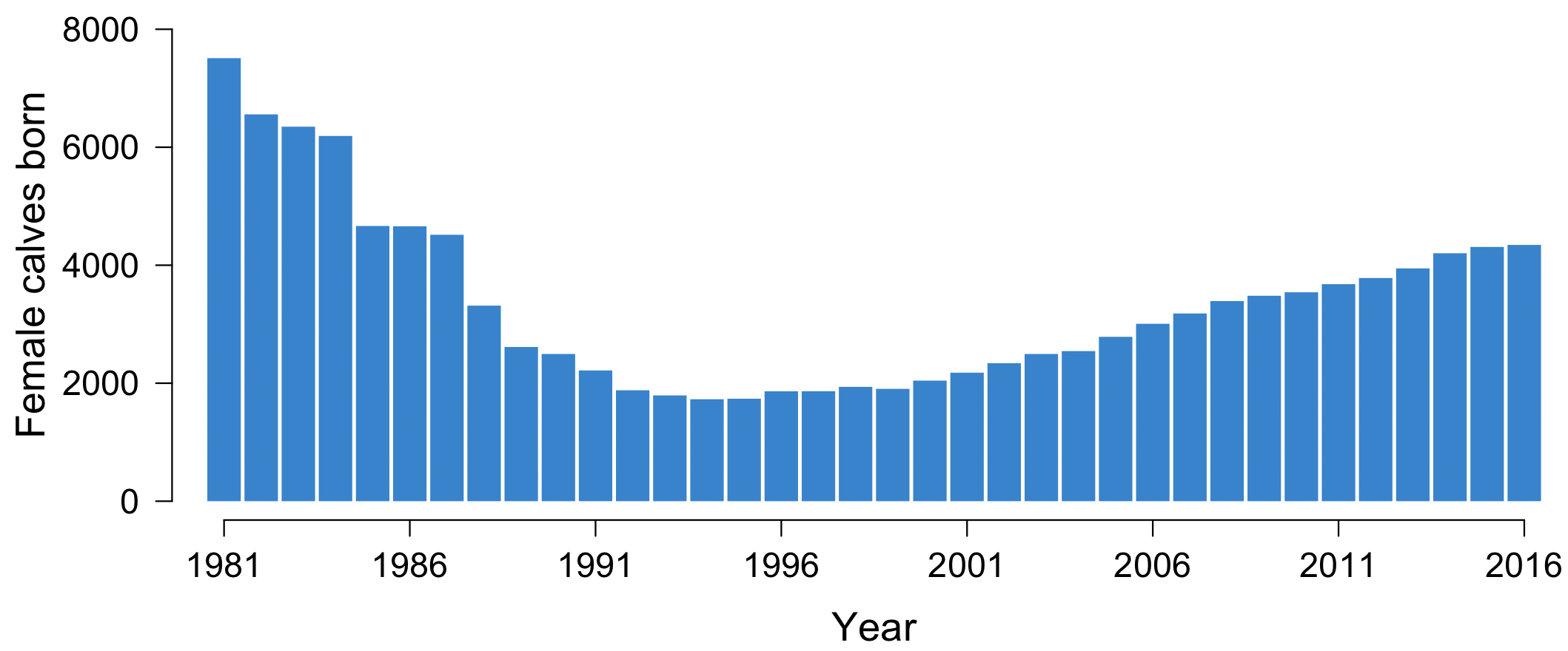

Supplement: Supplementary file 1 — Additional file 1: Original Braunvieh herd book population. Number of female calves entering the OB herd book between 1980 and 2016. [file 12864_2020_6446_MOESM1_ESM.docx]

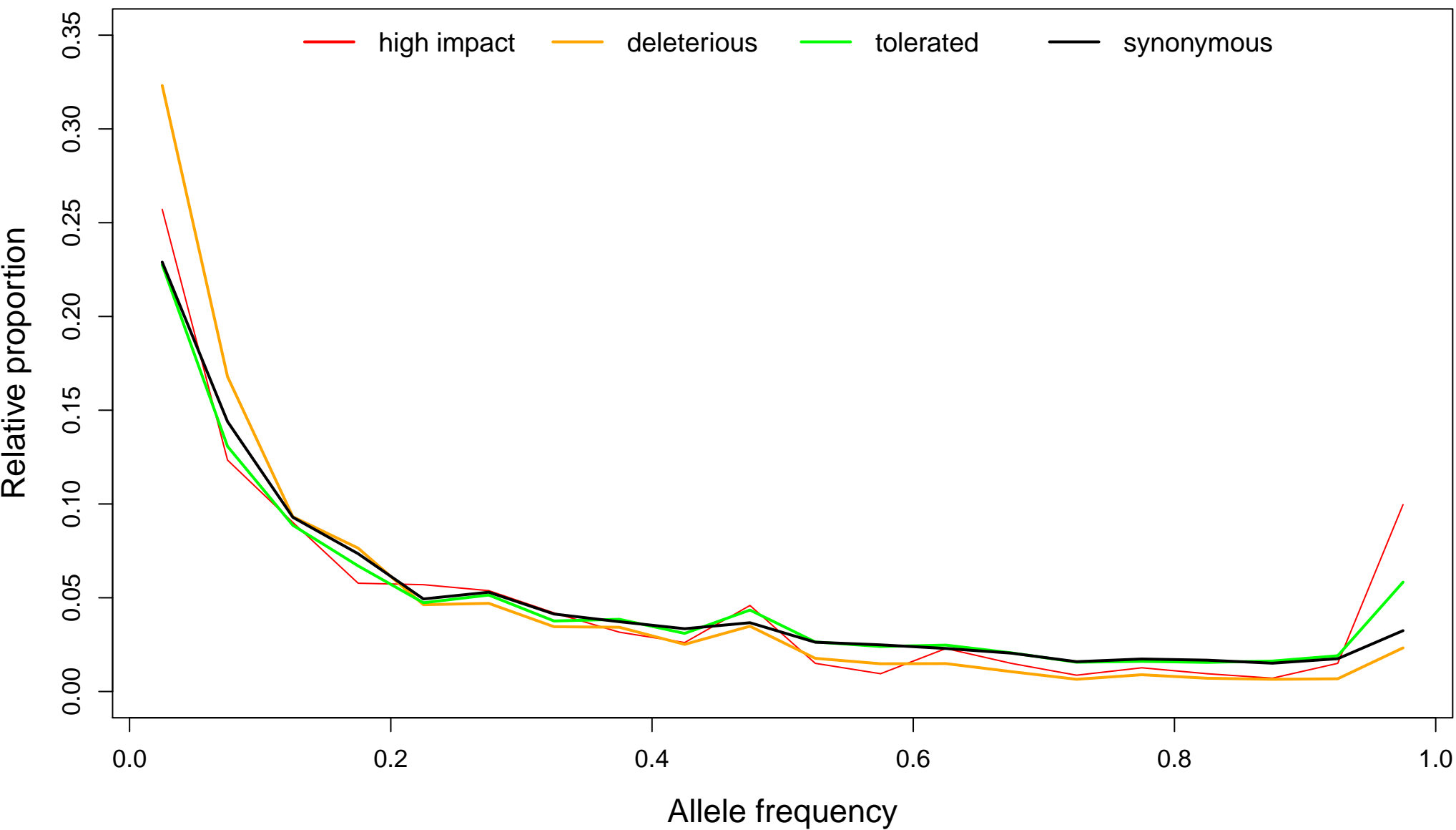

Supplement: Supplementary file 2 — Additional file 2: Allele frequency distribution in different functional annotations. Allele frequency of SNPs with different consequences according to VEP prediction, like high impact, deleterious (missense SNP with SIFT score < 0.05). tolerated (missense SNPs with SIFT score > 0.05) and synonymous SNPs. [file 12864_2020_6446_MOESM2_ESM.pdf]

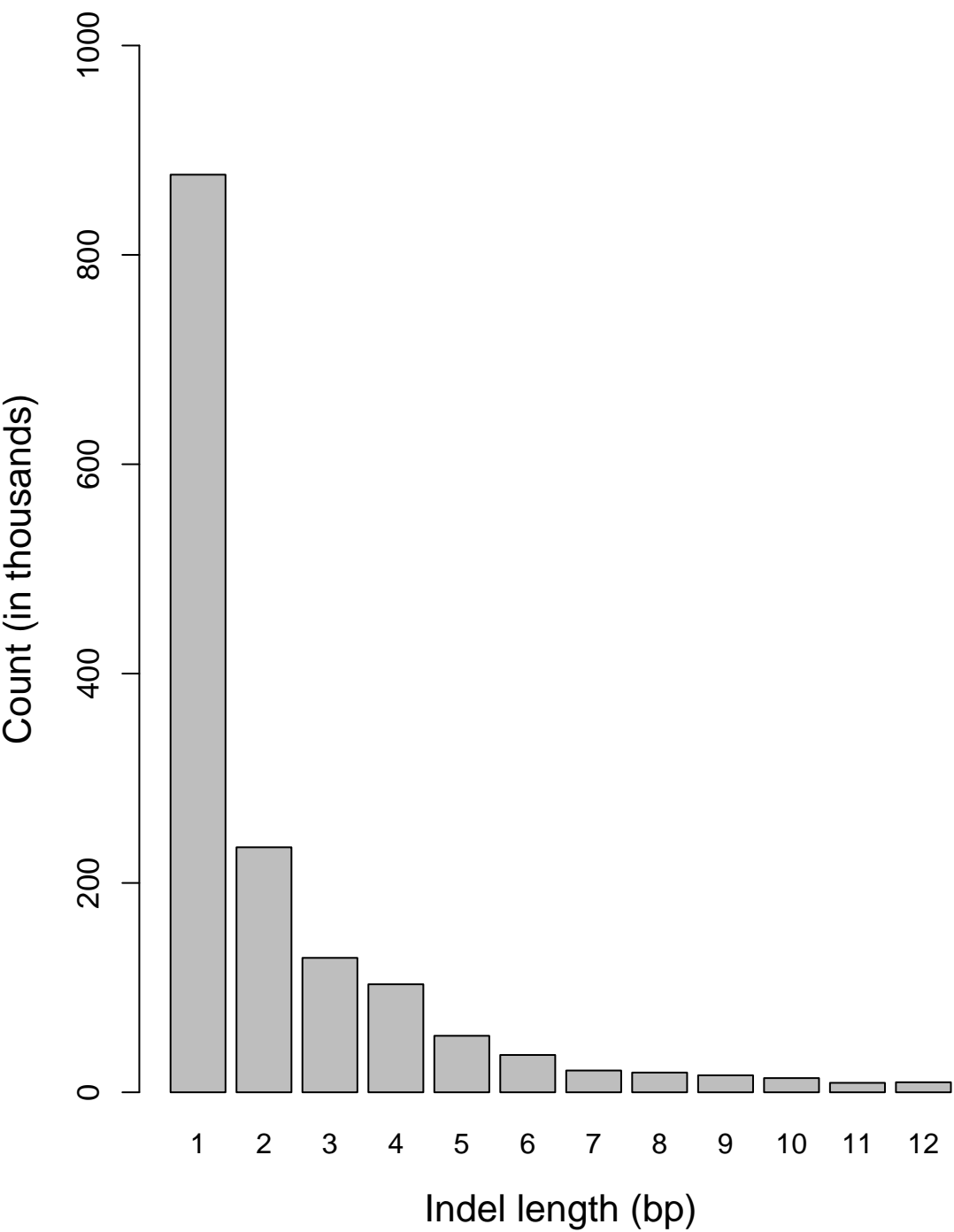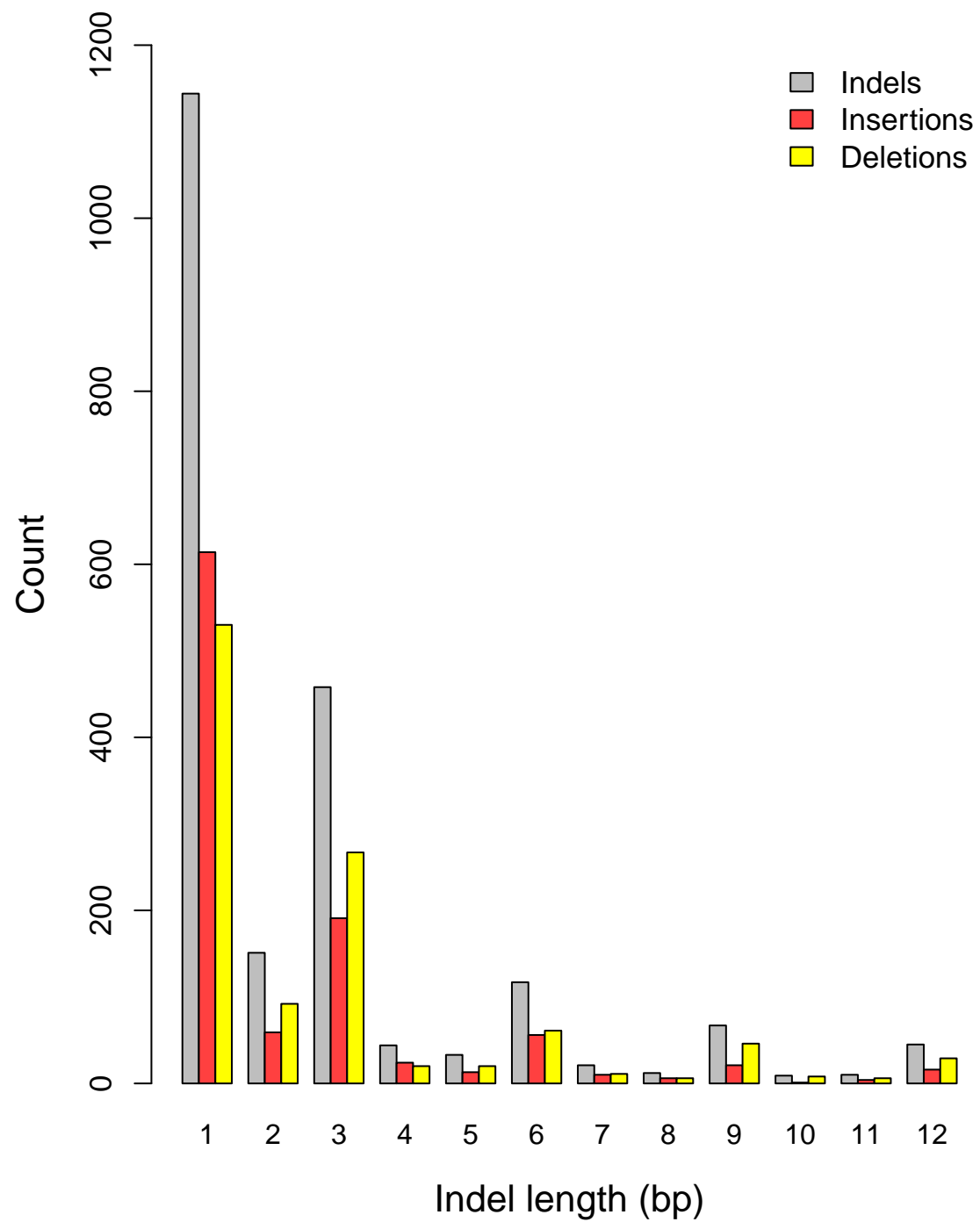

Supplement: Supplementary file 3 — Additional file 3: Distribution of length of Indels. a Number of Indels (× 1000) with size less than 12 bp detected according to the number of affected bases. b number of Indels detected in coding sequences. [file 12864_2020_6446_MOESM3_ESM.pdf]

Genomic inbreeding

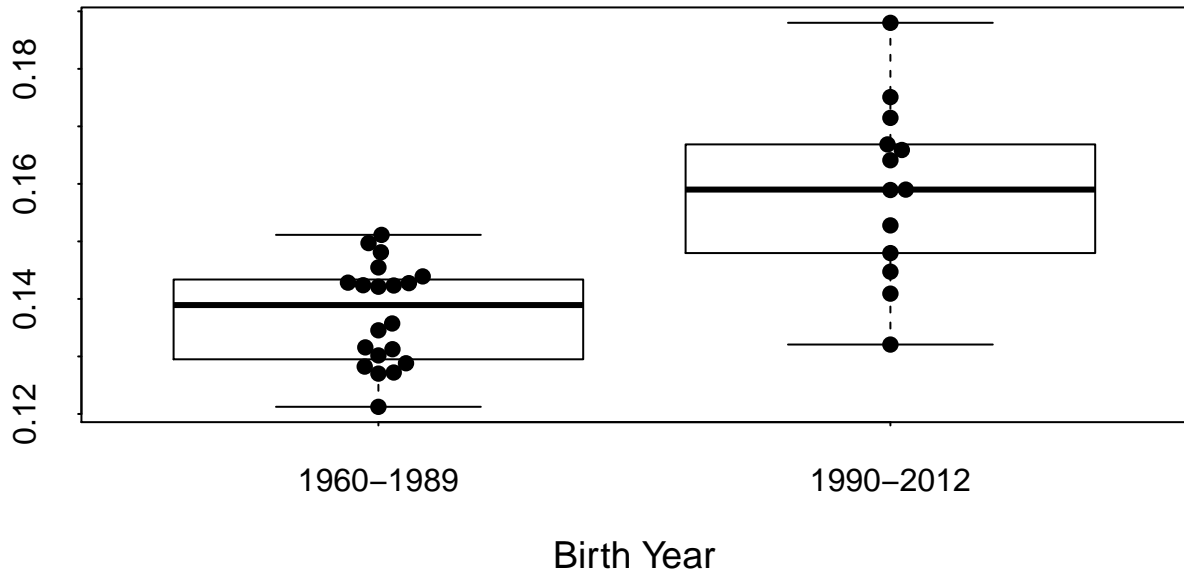

Supplement: Supplementary file 6 — Additional file 6: Genomic inbreeding in OB cattle stratified by birth year. Genomic inbreeding in two groups of animals born either between 1960 and 1989 or between 1990 and 2012. [file 12864_2020_6446_MOESM6_ESM.pdf]

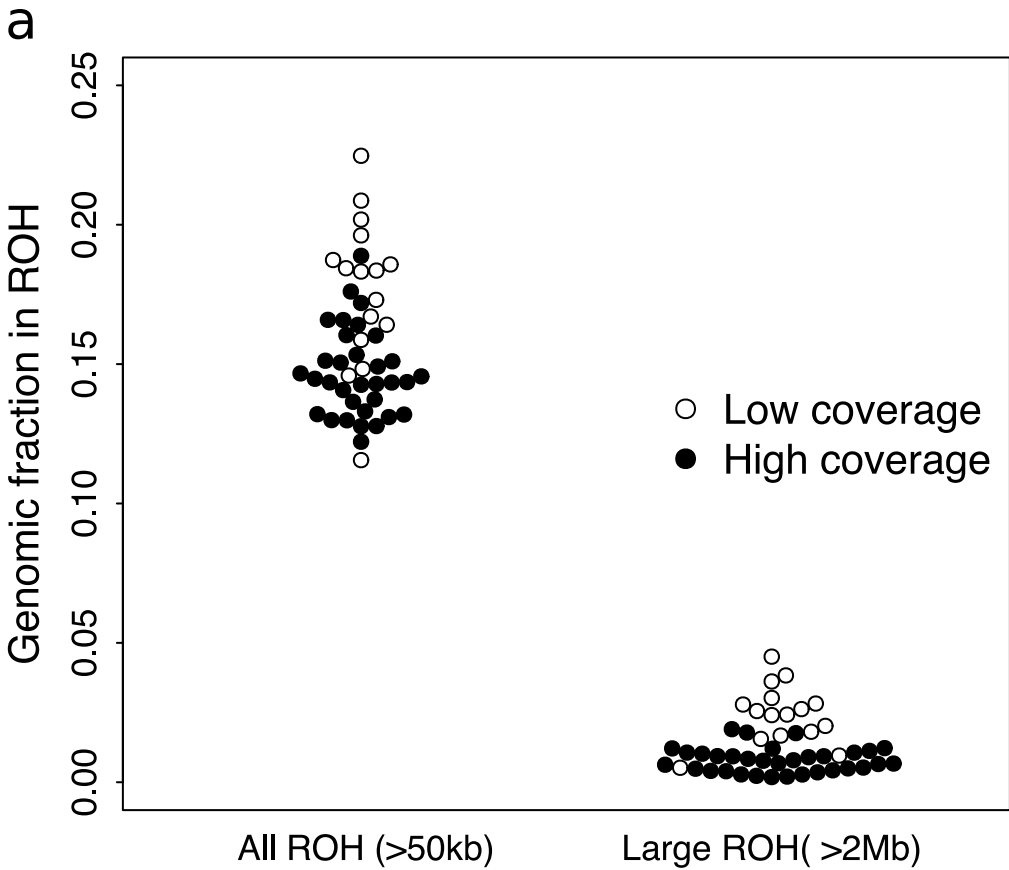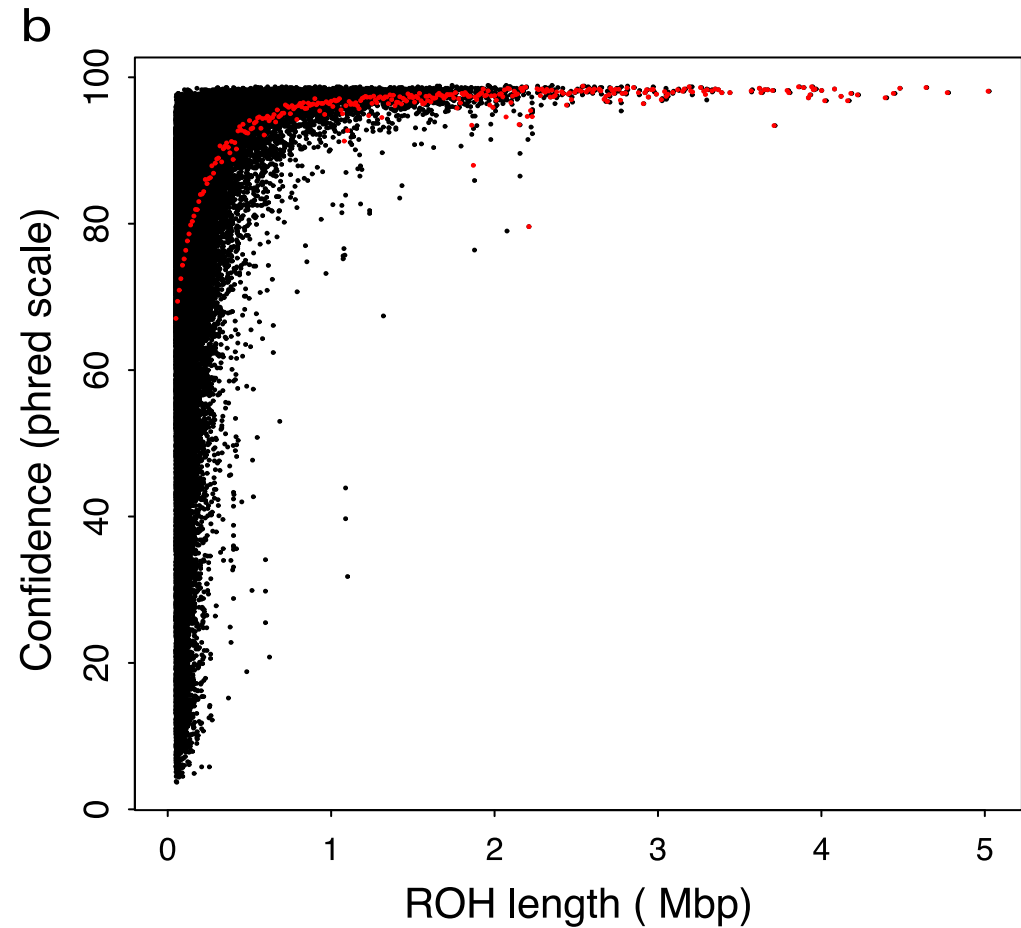

Supplement: Supplementary file 13 — Additional file 13: Runs of homozygosity in 49 OB cattle. a Total genome fraction in ROH in 49 cattle with high (>10x) and low (<10x) coverage (b) Phred confidence score for ROH in 33 cattle sequenced at average sequencing depth higher than 10-fold. Red dots indicate mean confidence scores for ROH. [file 12864_2020_6446_MOESM13_ESM.pdf]
